# A Case of Premature Ovarian Failure in a 33-Year-Old Woman

**DOI:** 10.1155/2013/573841

**Published:** 2013-01-29

**Authors:** Emma Colao, Teresa Granata, Marco F. M. Vismara, Francesco Bombardiere, Donatella Nocera, Elisa Luciano, Nicola Perrotti, Paola Malatesta

**Affiliations:** Medical Genetics Unit, University Hospital “Policlinico Mater Domini”, Magna Graecia University at Catanzaro, Viale Europa, Campus Salvatore Venuta, 88100 Catanzaro, Italy

## Abstract

*Objective*. To assess aetiology of a POF in a 33-year-old woman and, if possible, plan a cure. *Design*. Case report. *Setting*. medical genetics diagnostic unit in a university hospital. *Patient*. A 33-year-old woman with premature ovarian failure (POF). *Intervention(s)*. Genetic counseling, karyotyping, FISH study. *Result(s)*. Turner-like diagnosis. *Conclusion(s)*. Most cases of POF remain idiopathic. Turner syndrome can occur in very different phenotypes; cytogenetic and molecular profiling can provide a definitive diagnosis in cases with nonclassical phenotype.

## 1. Introduction

The average of age of the menopause is 50 years [1] with one percent of women menstruating after the age of 60 and one percent entering menopause before the age of 40.

Hereditary and environmental (smoking habit) factors affect the age of the natural menopause. A menopause before the age of 40 is commonly defined as “premature ovarian failure” although this definition is arbitrary. Estimates of the prevalence of premature ovarian failure range between 0.3 and 1% and this condition accounts for 10–28% of women with primary amenorrhoea and 4–18% of women with secondary amenorrhoea [2] (Table 1).

Some of the causes of POF listed above are extremely rare—particularly the genetic causes. For instance only a few women with FSH-receptor or LH-receptor mutations have ever been reported. In order to give an idea of how common various conditions are, here is a list of the causes of POF in women attending a specialist clinic at the Middlesex Hospital, London, UK (Table 2).

10 to 30% of women with POF already have a concurrent autoimmune disorder the most common of which is hypothyroidism. In the sequence of the most common autoimmune associations found in APSII, ovarian failure can occur at any stage. Thus, a history should include symptoms of Addison's disease, hypothyroidism, and diabetes in particular with the rarer association of autoimmune arthitides and inflammatory bowel disease being kept in mind. Further, women with established POF should be monitored for the later appearance of these conditions.

The only features of history which are helpful in determining aetiology of ovarian failure are positive a family history, a concurrent autoimmune disorder, or stigmata of one of the inherited conditions. In many instances a formal pedigree enquiry is required to determine other female family members who may be affected, particularly if the inheritance is passed through an unaffected male.

## 2. Case Presentation

A 33-year-old woman affected by hypergonadotropic hypogonadism (FSH 71.38 mIU/L; LH 35.50 mIU/L; estradiol <7 pg/mL) had been treated with estroprogestinic therapy “Triminulet” for nine years. Treatment was then suspended to allow a reevaluation of ovarian functionality. Upon drug withdrawal the patient presented secondary amenorrhea and was referred to our unit for an indepth diagnostic evaluation. A written informed consent was obtained.

Medical history revealed that the patient was born with normal delivery, bottle-fed, and showed standard psychosomatic development, menarche at age of 13 and oligomenorrhea since then up until estroprogestinic therapy was established. Pelvic US demonstrated a normally placed and normally developed uterus with a slightly dishomogeneous structure. The ovaries were not visible.

**Figure 1:**
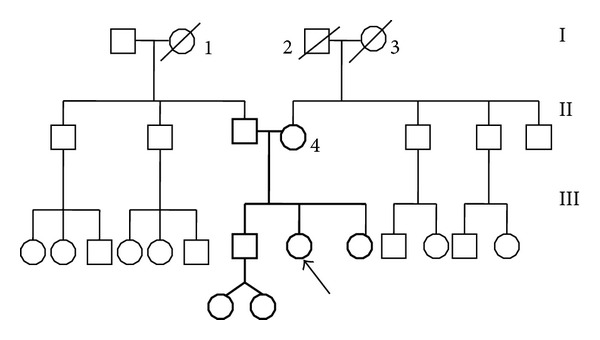
Pedigree of the patient: I, 1: deceased woman, 70 y.o., cardiac pathology I, 2: deceased man, 70 y.o., Parkinson's disease I, 3: deceased woman, 70 y.o., Type II diabetes II, 4: multinodular goitre.

The patient was also affected by autoimmune thyroiditis, treated with levotiroxin. Ten years earlier (2002) the patient received a diagnosis of lactotrophic microadenoma (PRL 125 ng/dL); brain MRI “dubious small area of enhancement in the lower right paramedian pituitary” and was treated with different dosages of cabergoline. This treatment was suspended in august 2010. In 2011 a brain MRI was substantially unmodified compared with with the previous one. In addition the patient reported myopia and arthralgia since she was 15 years old Family history showed familiarity for thyroid, neurological cardiovascular diseases (see pedigree: Figure 1).

Physical examination: height 158 cm (3°–25°p), weight 64 Kg (50°p), BMI 25,63 Kg/m^2^, waist circumference 92 cm, cranic, and upper and lower limb measurements between 50° and 75° percentile. No alterations detected in fingers and toes. Apparently a normal development was observed for secondary sex characteristics. Facial features revealed no dysmorphisms, moreover neither mental impairment or cognitive deficit was evident. Vital signs were normal.

**Figure 2:**
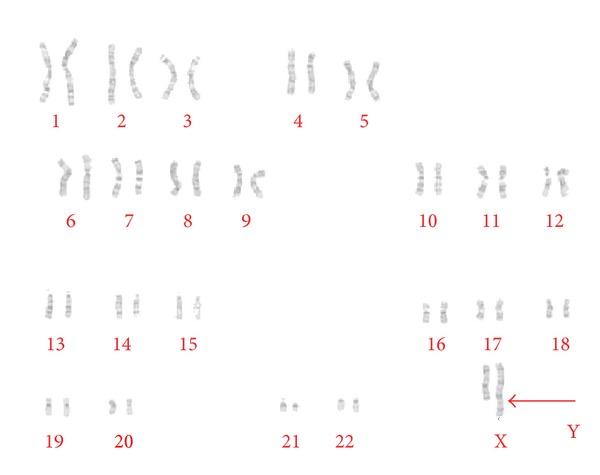
GTG Karyotype: X rearranged population [46,X,rea(X)].

A blood sample was drawn in Sodium (Na) heparin coated tube (vacutainer) for mononuclear cells culture and GTG karyotyping. High-definition GTG banding analysis of 100 metaphases (*Leica CW4000 *software) demonstrated a 45,X (41%)/46,X,rea(X) (59%) mosaicism (Figure 2).

**Figure 3:**
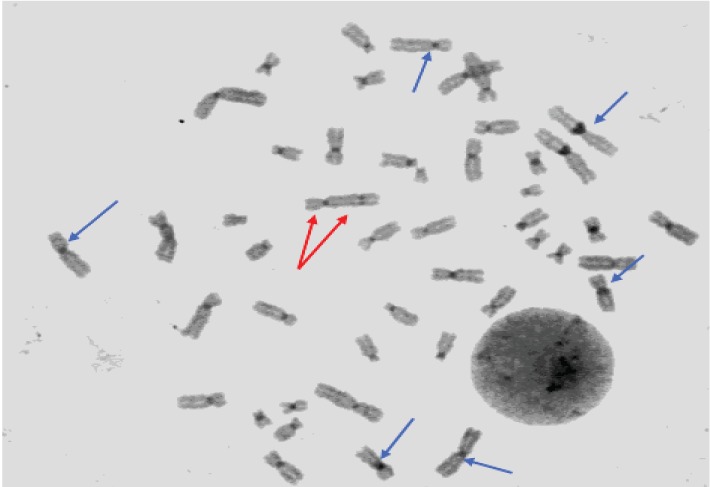
CBG Karyotype: red arrows show the two centromeres on dicentric X chromosome; blue arrows point to normal chromosomes (one centromere).

**Figure 4:**
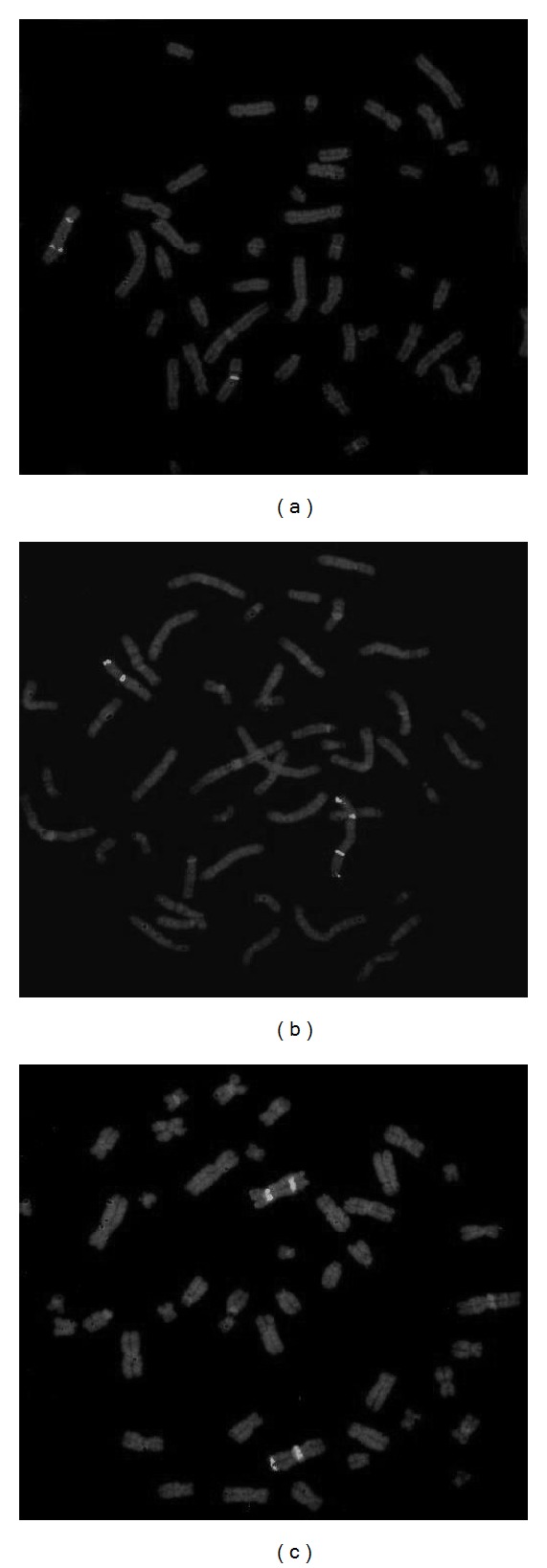
FISH images: (a) Aqua probe for X centromeres that demonstrates an X isodicentric subpopulation. (b) Two Orange/Green signals for Xp extremity on the isodicentric X (with two Aqua signals). (c) No Orange/Green signals for Xq in the isodicentric X (Aqua).

CBG staining of a new lymphocyte preparation demonstrated the presence of two centromeres in the X rearranged population [46,X,rea(X)] (Figure 3). Finally FISH analysis (kit *ToTelVysion (TM) Multicolor DNA probe mixtures (Vysis)*), confirmed the presence of two X centomeres in rearranged X chromosomes with 2 Xp extremities and no Xq extremity. In the rearranged X chromosome the Xq extremity was substituted by Xp and pericentrometric Xq region. After FISH analysis the subpopulation previously indicated as 46,X,rea(X) was then redefined as 46,X,idic(X)(q24) (Figure 4).

The final definition of the karyotype as released to the is **mos 46,X,idic(X)(q24)?[**59**]/45,X [**41**],** compatible with a “Turner like” condition that can account for the clinical and endocrinological condition of the patient.

After a written informed consent was obtained from first degree relatives of our patient (parents and sister), a GTG karyotype analysis extended to family members demonstrated that the phenotype observed in our patient was the result of a *de novo* alteration.

## 3. Discussion

Turner's syndrome accounts for most of the cases of nonidiopathic premature ovarian failure [3]. Turner's syndrome is a syndrome of defective gonadal development in phenotypic females mostly associated with the karyotype 45,X. Patients generally present short stature with gonadal dysgenesis (streak gonads), webbing of the neck, cubitus valgus, elevated gonadotropins, decreased estradiol level in blood, hypothyroidism, congenital heart defects, and primary amenorrhoea with failure to develop secondary sex characteristics (sexual infantilism) [4].

Mosaicisms accounts for 35% of Turner syndrome cases. 10% of these cases are due to mosaicisms for an X isochromosome [5]. Typically, in case of mosaicism, patients may present features that do not satisfy all the characteristics of the syndrome.

The case examined in the paper shows an attenuated phenotype, consistent with an X chromosome mosaicism. Indeed 41% cells have a monosomy of the X chromosome (45,X karyotype). The remaining 59% cells have an isodicentric X chromosome, then presenting a trisomy of Xp, monosomy of Xq24, and partial trisomy of the rest of Xq. 

This can explain the atypical phenotype of the patient: even in absence of classical dysmorphic features (*pterigio*, typical *facies*, and primary amenorrhea), she still suffers from other peculiar endocrine alterations, like hypergonadotropic hypogonadism, hypothyroidism, and autoimmune phenomena.

Regarding primary amenorrhea, this is not present in our case, although she has undergone to a premature ovarian failure.

Interestingly menarche and ovarian function are essentially regulated by two genes on X chromosome: POF1 and POF2, respectively, located on Xq24-Xq28 and Xq21.33. Based on our karyotype analysis we can say that, in the subpopulation of rearranged X chromosomes, POF1 results deleted, whereas POF2 is present in three copies.

We hypothesize that this peculiar genetic configuration can be sufficient to induce menarche, although POF1 haploinsufficiency is probably inadequate to sustain ovarian function thus leading to secondary amenorrhea.

Another trait typical of Turner's syndrome is short stature. In this case, patient's stature is in the lower normal range, included between the 3 and the 25 percentile. The position of short stature homeobox gene (SHOX gene) can explain, in part, this phenotypic feature.

The *SHOX* gene belongs to a family of genes called homeoboxes and is also part of the family of genes called PAR (Pseudoautosomal regions).

SHOX gene is made of seven exons, one of which is noncoding. Homeobox domain is coded by exons 3 and 4, that acts like transcriptional activators. SHOX codes also two alternatively spliced transcripts, **SHOXa **and **SHOXb**. The *SHOX* gene act during early embryonic development to control the formation of many body structures. Specifically, the *SHOX* gene is essential for the development of the skeleton and plays an important role in growth [6–8] and maturation of bones in the arms and legs.

**Figure 5:**
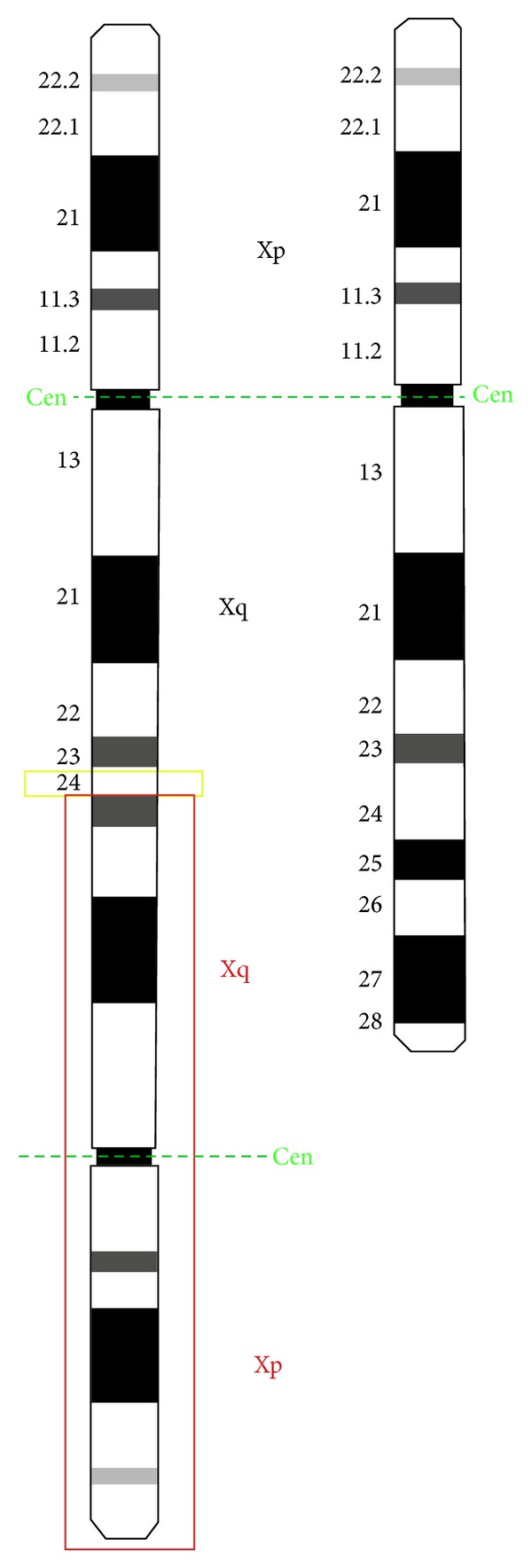
Comparison between reconstructedidiogram of the idic(X)(q24) and the normal X.

One copy of the *SHOX* gene is located on each of the sex chromosomes (the X and Y chromosomes) in an area called the pseudoautosomal region, Xp22.33 and Yp11.3 (Figure 5). Although many genes are unique to either the X or Y chromosome, genes in the pseudoautosomal region are present and are transcriptionally active on both chromosomes. As a result, females (who have two X chromosomes) and males (who have one X and one Y chromosome) have two functional copies of the *SHOX* gene in each cell.

Because the *SHOX* gene is located on the sex chromosomes, most women with Turner syndrome have only one copy of the gene in each cell instead of the usual two copies. Loss of one copy of this gene reduces the amount of SHOX protein that is produced. A shortage of this protein likely contributes to the short stature and skeletal abnormalities [9] often seen in females with Turner syndrome [10].

In the case described in the present paper, SHOX gene is triplicated in the cell subpopulation harboring the rearranged X chromosome and this triplication can probably in part correct the haploinsufficiency that is present in the 45,X cells [11] thus mitigating the short stature phenotype that is typical of Turner syndrome.

## Figures and Tables

**Table 1:** Causes of premature ovarian failure.

Gonadal dysgenesis	
Turner's syndrome	
Perrault's syndrome	
46, XX gonadal dysgenesis/46, XY gonadal dysgenesis	
Genetic associations	
Familial ovarian failure	
Galactosaemia	
Enzyme defects—P450c17	
FRAXA premutations	
Blepharophimosis, ptosis, and epicanthus inversus syndrome (also called BPES)	
Small X chromosome defects	
FSH receptor mutations/LH receptor mutations	
	
Autoimmune:	
Autoimmune Polyendocrinpathy syndrome 1	
APECED/autoimmune Polyendocrinpathy syndrome 2	
Association with various autoimmune diseases	
Isolated autoimmune ovarian failure	
	
Iatrogenic chemotherapy	
Radiotherapy	
Pelvic surgery	
Environmental toxins	
Idiopathic?	

**Table 2:** Causes of POF in women attending a specialist clinic at the Middlesex hospital, London, UK from [2].

Diagnosis	n°
Idiopathic POF (no cause found)	245
Turner's syndrome	162
Autoimmune polyendocrinopathy type 2	45
Familial POF	36
Galactosaemia	24
FRAXA premutations	7
BPES	3
X chromosome breakpoints	3
APS 1/APECED	2
Perrault's syndrome	1
